# DNA Damage-Inducible Transcript 4 Is an Innate Surveillant of Hair Follicular Stress in Vitamin D Receptor Knockout Mice and a Regulator of Wound Re-Epithelialization

**DOI:** 10.3390/ijms17121984

**Published:** 2016-11-26

**Authors:** Hengguang Zhao, Sandra Rieger, Koichiro Abe, Martin Hewison, Thomas S. Lisse

**Affiliations:** 1 Department of Dermatology, The First Affiliated Hospital of Chongqing Medical University, Chongqing 400016, China; 2 Kathryn W. Davis Center for Regenerative Biology and Medicine, Mount Desert Island Biological Laboratory, 159 Old Bar Harbor Road, Salisbury Cove, ME 04672, USA; srieger@mdibl.org; 3 Department of Molecular Life Science, Tokai University School of Medicine, Isehara, Kanagawa 259-1193, Japan; abeko@is.icc.u-tokai.ac.jp; 4 Institute of Metabolism and Systems Research, College of Medical and Dental Sciences, The University of Birmingham, Birmingham B15 2TH, UK; m.hewison@bham.ac.uk; 5 The Jackson Laboratory, Bar Harbor, ME 04609, USA

**Keywords:** wound repair, VDR, mTOR, DDIT4, stress, hair follicle, stem cells

## Abstract

Mice and human patients with impaired vitamin D receptor (VDR) signaling have normal developmental hair growth but display aberrant post-morphogenic hair cycle progression associated with alopecia. In addition, VDR^–/–^ mice exhibit impaired cutaneous wound healing. We undertook experiments to determine whether the stress-inducible regulator of energy homeostasis, DNA damage-inducible transcript 4 (Ddit4), is involved in these processes. By analyzing hair cycle activation in vivo, we show that VDR^−/−^ mice at day 14 exhibit increased Ddit4 expression within follicular stress compartments. At day 29, degenerating VDR^−/−^ follicular keratinocytes, but not bulge stem cells, continue to exhibit an increase in Ddit4 expression. At day 47, when normal follicles and epidermis are quiescent and enriched for Ddit4, VDR^−/−^ skin lacks Ddit4 expression. In a skin wound healing assay, the re-epithelialized epidermis in wildtype (WT) but not VDR^−/−^ animals harbor a population of Ddit4- and Krt10-positive cells. Our study suggests that VDR regulates Ddit4 expression during epidermal homeostasis and the wound healing process, while elevated Ddit4 represents an early growth-arresting stress response within VDR^−/−^ follicles.

## 1. Introduction

Besides its known regulation of mineral homeostasis, the vitamin D receptor (VDR) also plays a functional role within the cutaneous environment. The importance of the cutaneous VDR is demonstrated in certain human patients with hereditary vitamin D-resistant rickets and mice that harbor loss-of-function mutations in the VDR and eventually develop hair loss (alopecia totalis [1]), as well as impaired skin wound repair [2,3]. In utero, the epithelium and underlying mesenchyme interact to form hair follicles during morphogenesis, which ends during the second week of life in mice. During the ensuing post-morphogenic hair cycle, the permanent follicular component is retained, which includes sebaceous glands and the upper outer root sheath that harbors the hair follicle stem cell center called the bulge. In contrast, the lower part of the follicle cycles through episodic periods of growth, regression and quiescence, termed the anagen, catagen and telogen stages respectively. The telogen-to-anagen transition marks a stage in which stem cells become activated to form a new hair bulb with concomitant inner root sheath (IRS) and hair shaft differentiation.

The VDR is thought to play a limited role during the early morphogenic period of follicular development since VDR^−/−^ mice appear grossly normal and generate their first coat of hair [4]. However, VDR^−/−^ mice exhibit compromised post-morphogenic hair cycles and improper anagen initiation and integrity, in which mutant mice do not grow new hair, and instead the follicles become epidermal cysts [5,6,7]. The signaling defects within VDR-deficient hair follicles are localized to keratinocytes, and not to the mesenchymal component of the follicle [6,7,8]. Through a series of recovery transgenesis experiments in mice, studies have shown that the zinc finger DNA binding domain of the VDR, but not the ligand binding or activation function 2 domains, was critical for the hair cycle [9,10,11].

Despite these studies, the biological explanation for hair loss in VDR-deficient mice remains unclear. The VDR was identified as a major molecular signature within hair bulge label-retaining stem cells [12], with subsequent studies suggesting that unliganded VDRs are required for the self-renewal, colony formation and normal lineage specification, as well as function of bulge keratinocyte stem cells (KSCs; [13]). However other follow-up studies observed no functional defects in VDR-deficient KSCs [14]. Hair follicle induction and maintenance are controlled by numerous factors such as fibroblast growth factor, transforming growth factor beta, Hedgehog and Wnt signaling, by controlling the self-renewal and lineage progression of KSCs and activation of progenitor cells. Studies suggest that VDR interactions with the lymphoid enhancer-binding factor 1 (lef1) transactivator may be the mechanism by which the unliganded VDR promotes Wnt signaling [13,15]. Importantly, it was shown that the VDR interaction with the canonical Wnt target gene, *Axin2*, was perturbed in both VDR-null and Lef1-null keratinocytes [16]. Furthermore, the Hedgehog signaling pathway was found to be disrupted in VDR- and Lef1-null keratinocytes, thus contributing to defective follicular signaling [16,17].

Besides its role in hair follicle biology, the VDR is expressed ubiquitously in the basal layer of the interfollicular epidermis [14], and plays a role in epidermal integrity and wound repair. In the epidermis, the biologically active form of vitamin D, 1,25-dihydroxyvitamin D (1,25D_3_), is known to suppress tumor formation, promote innate antimicrobial immunity, suppress epidermal keratinocyte proliferation and promote differentiation and formation of the permeability barrier (reviewed in [18]). Epidermal abnormalities are observed in VDR^−/−^ mice at as early as one week of age, with age-dependent reduction of differentiation markers and lipid content [4,19,20]. Consequently, the epidermis of VDR^−/−^ mice is generally thicker and more folded, reflecting enhanced epidermal proliferation [21]. In contrast to VDR^−/−^ mice, mice lacking the enzyme that catalyzes synthesis of 1,25D_3_ (cytochrome p450 27B1; CYP27B1^−/−^) have normal hair, but altered epidermal differentiation signatures, impaired permeability barrier and wound healing responses, suggesting that 1,25D_3_ is crucial for epidermal integrity [22,23]. The cutaneous wound healing defects in global VDR^−/−^ animals have been characterized by scarcity of infiltrating macrophages in the early inflammatory phase and disrupted vascular invasion of granulation tissue, yet re-epithelialization was reported to be unaffected [2]. In contrast, epithelial-specific deletion of the VDR in mice results in impaired re-epithelialization of wounds [3]. Also, in vitro scratch migration assays using VDR^−/−^ primary keratinocytes showed decreased injury-stimulated migration [14].

Mechanistic/mammalian target of rapamycin (mTOR) is a serine-threonine protein kinase that is considered to be a “master regulator” of cell growth and survival. It is the major checkpoint for sensing of cellular nutrients and oxygen and energy levels to orchestrate the cellular responses to any changes. Upstream inhibitors of mTOR have been characterized, including DNA damage-inducible transcript 4 (Ddit4), also called REDD1, a mitochondrial protein which is involved in the conservation of energy resources. *Ddit4* was first identified as a stress response gene transcriptionally activated upon UV-induced DNA damage [24], and then during hypoxia, oxidative stress, endoplasmic reticulum (ER) stress, atrophied glucocorticoid treatment and energy/serum deprivation [25,26,27,28]. We have recently shown that *Ddit4*/*DDIT4* is a direct and conserved transcriptional target of the VDR in human and mouse bone-forming osteoblasts to promote differentiation [29,30]. In view of the ubiquitous expression pattern of Ddit4 within the mammalian system, the present study focused on potential cutaneous interactions between VDR and Ddit4 signaling. Accordingly we hypothesize that in mice: (1) *Ddit4* is functionally regulated by the VDR during the hair cycle and epidermal wound repair, and (2) given its known role as a stress-inducible factor, Ddit4 may be utilized as an innate surveyor of VDR-dependent adverse cellular effects. By monitoring the effects on Ddit4 we were able to show that the VDR and Ddit4 function distinctly at the crossroads between hair follicle cycling and the epidermal wound repair process.

## 2. Results

### 2.1. DDIT4/Ddit4 Is an Acute Phase Effector of Stress Related to Inflammatory and Other Immune Challenges to Physical or Bacterial Complications

**Figure 1:**
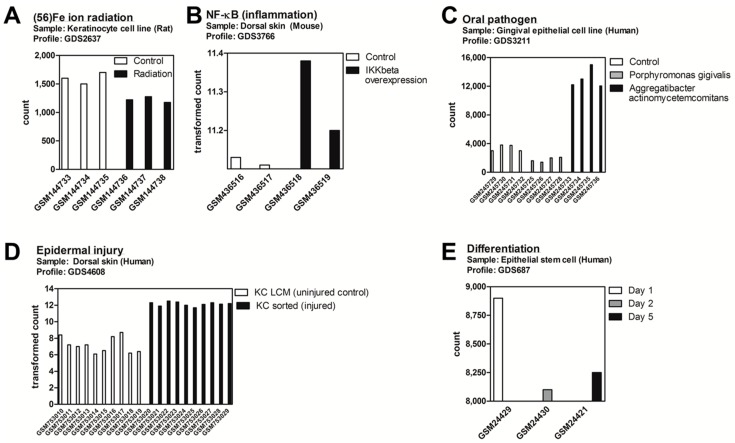
Gene Expression Omnibus (GEO) profile assessment of *Ddit4*/*DDIT4* responses to biological and stress conditions within epithelial cells. (1.5 column) (**A**) For all DataSets presented in this figure, the graphs represent *Ddit4*/*DDIT4* transcript levels. DataSet GDS2637 shows that *Ddit4* was not induced after 56FE ion irradiation within a rat keratinocyte cell line; (**B**) DataSet GDS3766 shows that NF-κB activation by IKKβ overexpression in mouse dorsal skin resulted in increased *Ddit4* levels; (**C**) DataSet GDS3211 reports that periodontal pathogen infection of a human gingival epithelial cell line with *Aggregatibacter actinomycetemcomitans* led to *DDIT4* induction; (**D**) DataSet GDS4608 shows that upon injury of human epidermal cells, *DDIT4* transcript levels were increased compared to uninjured samples; (**E**) DataSet GDS687 shows that differentiation of keratinocyte stem cells upon mesenchymal-epithelial interactions resulted in decreased *DDIT4* expression over time. All DataSets can be accessed at: http://www.ncbi.nlm.nih.gov/geoprofiles.

We initially sought a general assessment under which biological stresses regulate *DDIT4*/*Ddit4* gene expression within keratinocytes and other epithelial cell types. To do this, we appraised the publically available Gene Expression Omnibus (GEO) Profiles repository of curated microarray/next generation sequencing datasets for individual gene expression profiles (available at: http://www.ncbi.nlm.nih.gov/geoprofiles). We identified five (cutaneous) experimental DataSet records whereby *DDIT4*/*Ddit4* was significantly regulated compared to control samples. In a study to explore the space effects of heavy Fe ion radiation exposure of a rat keratinocyte line, *Ddit4* mRNA expression was decreased, despite the induction of other “DNA repair” genes within the data set [31] and original discovery of *Ddit4* as a major transcript activated by UV irradiation [24] (Figure 1A). In contrast, under the stress of inflammation (i.e., via overexpression of the NF-κB activator IKKβ) [32] or specific types of periodontal pathogens [33], *Ddit4/DDIT4* transcript was increased within mouse skin and human gingival epithelial cells, respectively (Figure 1B,C). In another study, human keratinocytes that were exposed to an enzymatic cell disassociation treatment as a model of epidermal injury [34], resulted in elevated *DDIT4* message (Figure 1D). In a more unperturbed physiological setting, multipotent human hair follicle stem cells were co-cultured with mesenchymal dermal papilla cells to induce keratinocyte progenitor differentiation, whereby *DDIT4* message levels decreased over time [35].

### 2.2. VDR Positively Regulates Ddit4 mRNA and Protein Expression in Primary Murine Epidermal Keratinocytes

**Figure 2:**
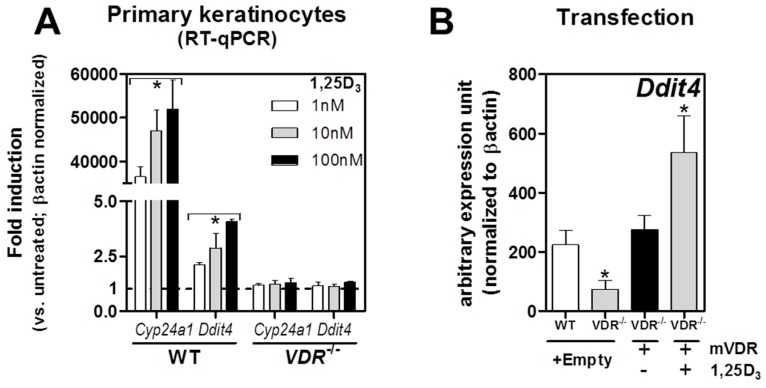

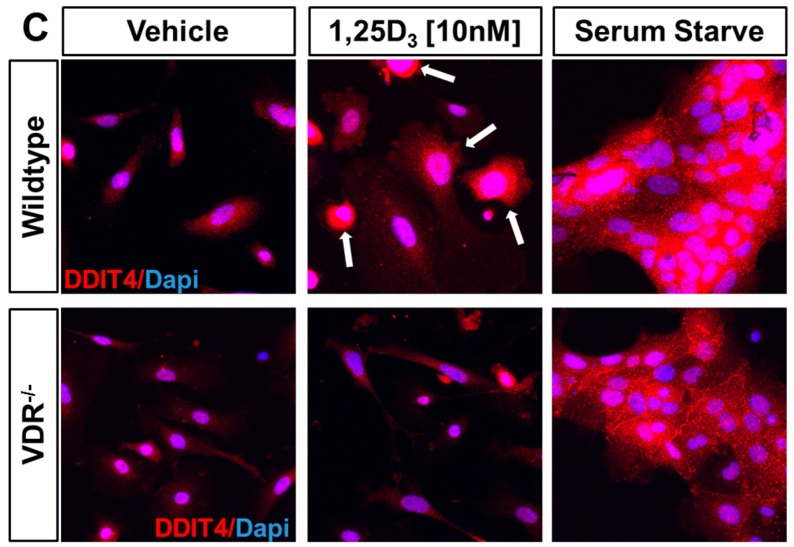
Regulation of the mechanistic/mammalian target of rapamycin (mTOR) inhibitor and stress sensor DNA damage-inducible transcript 4 (Ddit4) by liganded vitamin D receptors (VDRs) within primary keratinocytes (column 1.5). (**A**) Dose-dependent transcriptional induction of *Cyp24a1* and *Ddit4* by 1,25-dihydroxyvitamin D (1,25D_3_, 6 h) in wildtype (WT), but not VDR^−/−^, primary keratinocytes derived from neonatal pups; (**B**) Transient transfection of VDR^−/−^ primary keratinocytes with a mVDR plasmid restores endogenous *Ddit4* transcript levels. Combined treatment further enhanced *Ddit4* mRNA levels (with mVDR transfection and 1,25D_3_ of 10 nM for 6 h); (**C**) Immunofluorescence detection of Ddit4 within primary keratinocytes. WT, but not VDR^−/−^, primary keratinocytes exposed to 10 nM 1,25D_3_ for 18 h resulted in increased intracellular accumulation of Ddit4 (white arrows). Ddit4 upregulation in response to fetal bovine serum deprivation for 24 h in both WT and VDR^−/−^ keratinocytes. One-way ANOVA at an α = 0.05 (95% confidence interval) and Tukey’s multiple comparison post-tests were utilized. Significance is denoted with asterisks: * *p* < 0.05 (*n* = 3–4 experiments). RT-qPCR: reverse transcription quantitative polymerase chain reaction.

To investigate the VDR-Ddit4 relationship within the mouse cutaneous system, we first determined whether 1,25D_3_-VDR actions could transcriptionally activate *Ddit4* within primary epidermal keratinocytes harvested and cultured from neonatal pups aged 2–3 days derived from either wildtype (WT) or VDR^−/−^ mice on a normalized calcium/phosphorous diet. Since keratinocytes differentiate with calcium, our culture system was established using low-calcium conditions (0.05 mM) to mimic undifferentiated cells in the basal layer that have the highest concentration and effectiveness of the VDR [36]. Treatment of WT, but not VDR^−/−^ cells with 1,25D_3_ (1–100 nM) for 6 h resulted in a dose-dependent induction of both *cyp24a1* (1,25-dihydroxyvitamin D_3_ 24-hydroxylase), a major transcriptional target and negative feedback regulator of vitamin D, and *Ddit4* (Figure 2A). Expression of *Ddit4* mRNA levels in primary keratinocytes was significantly lower in VDR^−/−^ mice, but transfection of these cells with a murine Vdr transgene resulted in re-expression of *Ddit4*, and enhanced induction after 1,25D_3_ treatment (Figure 2B). These results demonstrate that *Ddit4* is directly controlled by VDR actions.

Next we investigated the effects on Ddit4 protein expression within WT and VDR^−/−^ primary keratinocytes using immunofluorescence staining. In WT cells treated with 1,25D_3_ (10 nM) for 18 h in growth medium supplemented with FBS with reduced-calcium (0.05 mM), we observed an increased number of cells with elevated intracellular accumulation of Ddit4 (Figure 2C, upper middle panel). In addition, these 1,25D_3_-treated WT keratinocytes appeared to be morphologically distinct (i.e., enlarged and differentiated) compared to their VDR^−/−^ counterparts. The 1,25D_3_-treated VDR-null cells appear to acquire higher length:width ratios, indicative of an undifferentiated motile state (Figure 2C, lower middle panel). Likewise, after 1,25D_3_ treatment of VDR-null keratinocytes, there was a comparable decrease in Ddit4 expression to that of WT cells. As a safeguard for energy conservation, Ddit4 expression is known to be enhanced under stress conditions such as growth factor (serum) deprivation [25]. With this in mind, we tested this response in WT keratinocytes and observed increased Ddit4 protein expression after serum starvation (Figure 2C, upper right panel). Interestingly, Ddit4 expression was also enhanced in VDR^−/−^ keratinocytes under serum-deprived conditions (Figure 2C, lower right panel). These results suggest that (1) the Ddit4-mediated energy and growth factor depletion sensing mechanism has the potential to function independent of cellular VDRs; and (2) endogenous Ddit4 activation can be used as an innate surveillant for catabolic stress within the VDR loss-of-function system.

### 2.3. Ddit4 Is a Direct Transcriptional Effector of the Liganded VDR within Primary Epidermal Keratinocytes

Transcriptional regulation by 1,25D_3_ involves occupancy of VDREs on effector genes by 1,25D_3_-bound or unbound VDRs and numerous other co-regulators. Here, we performed chromatin immunoprecipitation (ChIP) qPCR assays to assess potential VDRs and general transcriptional element binding to the *Ddit4* promoter within WT neonatal primary keratinocytes. We tiled the proximal promoter region (i.e., with primers Ddit4-1 and Ddit4-2), which was approximately 500 base pairs upstream of the *Ddit4* transcriptional start site (TSS) on the reverse strand. The distal region was appraised using primer Ddit4-3, which targeted a DNA region 1.7 kilobases upstream of the TSS. All sequence positions were based on the primary assembly GRCm38 released by the Genome Reference Consortium in 2012 based on the *Mus musculus* strain C57BL/6J.

**Figure 3:**
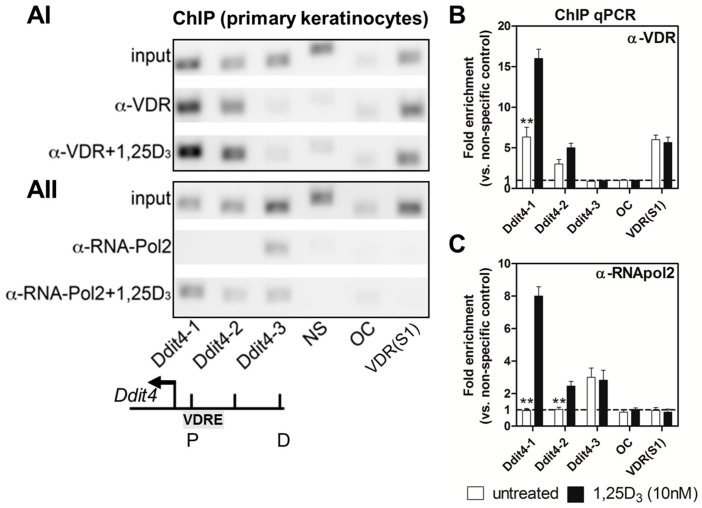
*Ddit4* transcriptional activity is directly regulated by the VDR within primary epidermal keratinocytes (1.5 column). (**A**) Representative chromatin immunoprecipitation (ChIP) gel images of VDR and RNA polymerase 2 (RNApol2) interactions at the *Ddit4* promoter region within WT primary keratinocytes. Figure 3(AI) shows VDR immunoprecipitation, while Figure 3(AII) shows RNApol2 immunoprecipitation results. Cells were treated with 1,25D_3_ (10 nM) for 15 min, fixed and then the chromatin was purified. NS (non-specific), OC (osteocalcin), and VDR-S1 (VDR intron 3 and 4) genomic sites were used for control purposes. P (proximal), D (distal), VDRE (putative vitamin D response element); (**B**) ChIP-Quantitative PCR (qPCR) analysis of VDR immunoprecipitation; (**C**) ChiP-qPCR analysis of RNApol2 immunoprecipitation. One-way ANOVA at an α = 0.05 (95% confidence interval) and Tukey’s multiple comparison post-tests were utilized. Significance is denoted with asterisks: ** *p* < 0.01 (*n* = 4 experiments).

ChIP results show that endogenous VDRs bound strongly to the proximal promoter of *Ddit4* under unstimulated conditions (Figure 3(AI)). Upon 1,25D_3_ stimulation (10 nM, 15 min) there was increased recruitment of liganded VDRs to the proximal, but not distal, promoter region of *Ddit4* as assessed by ChIP-qPCR (Figure 3B). These results suggest that the promoter region of *Ddit4* in keratinocytes includes functional VDRE(s). Although the unliganded VDR bound to the proximal *Ddit4* promoter, this represented a transcriptionally inactive state as RNA polymerase 2 (RNApol2), a general marker for precursor RNA synthesis, was tethered or poised at the more distal region (Figure 3(AII)). In contrast, there was accompanying RNApol2 binding activity at the proximal *Ddit4* promoter only after 1,25D_3_ treatment, suggesting direct transcriptional activation of *Ddit4* after ligand stimulation (Figure 3C). For ChIP validation we utilized the murine osteocalcin gene, *Bglap*, which has no VDREs in its promoter region, nor is it transcriptionally influenced by addition of 1,25D_3_ [37]. We also chose the S1 site within intron 3 and 4 of the murine *Vdr* gene as a potent positive control for both auto-regulatory VDR and RNApol2 binding, as previously shown in MC3T3-E1 murine osteoblasts [38]. Interestingly in primary keratinocytes, VDRs but not RNApol2, bound to the S1 site, suggesting cell type-specific usage of this transcriptional auto-regulatory site. All ChIP-qPCR results were normalized to a validated non-specific (NS) “naked” control target site . Overall, our findings support the notion that the liganded VDR directly targets the *Ddit4* promoter to induce transcription within primary epidermal keratinocytes.

### 2.4. Formation of Ddit4-Positve Stress Compartments in VDR^−/−^ Morphogenic Follicles and Reduced Ddit4 Epidermal Expression

**Figure 4:**
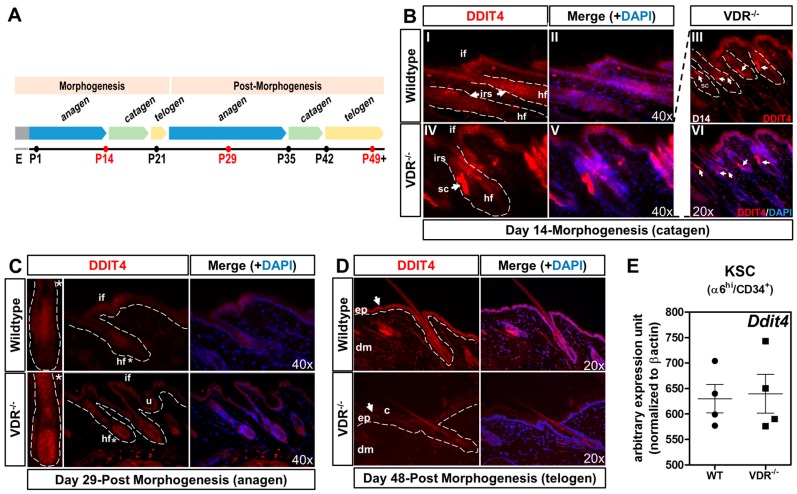
Ddit4α-positive stress compartments present in VDR^−/−^ morphogenic hair follicles (column 2). (**A**) Chart depicting the morphogenic and post-morphogenic stages of the hair cycle up to post-natal day 49 (P49); (**B**) At day 14, hair follicles are within the first postnatal morphogenic early catagen (regression) phase. In WT (**I**,**II**) and VDR^−/−^ (**IV**,**V**) animals, Ddit4 is expressed throughout the inner root sheath (irs), but not in the bulb of the hair follicle (hf). In both genotypes, Ddit4 was expressed within the interfollicular (if) epidermis. Hair follicles of VDR^−/−^ animals exhibit Ddit4-positive stress compartments (sc, white arrows) (**IV**). In the right panel (**III**,**VI**), a lower magnification (20×) is presented where each hair follicle is outlined with a white dotted line. Ddit4 immunostaining is counter-detected with Alexa^®^ 594 and nuclei stained with DAPI; (**C**) At day 29, hair follicles are within the second postnatal anagen (growth) phase. In WT skin, Ddit4 expression was attenuated within the IF and hair follicle compared to day 14. Asterisks reflect the zoom of the respective hair follicle. In VDR^−/−^ hair follicles, there was aberrant and increased Ddit4 expression throughout the length of the follicle (bulb to inner root sheath) compared to WT. There was a similar expression pattern in the utricles (u) and the interfollicular epidermis; (**D**) At day 48, WT skin display telogen hair follicles with quiescent morphology. Ddit4 expression was enhanced and uniform throughout the epidermis (**ep**) (white arrow), dermis (**dm**) and follicular epithelium. In contrast, VDR^−/−^ skin exhibited significant reduction of Ddit4 throughout the skin. c (corneocytes). For immunostaining, representative slides are presented. Dotted white lines outline individual hair follicles or the epidermal-dermal junction; (**E**) Hair follicle bulge stem cells from 29-day-old animals were fluorescence-activated cell sorting (FACS) purified and analyzed with RT-qPCR. There was no significant change in *Ddit4* message level between WT and VDR^−/−^ bulge stem cells (*n* = 4 experiments per genotype).

Having shown a direct transcriptional relationship between the VDR and *Ddit4* within primary keratinocytes, we next sought to decipher the potential in vivo role of Ddit4 during activation and resting stages of the hair follicle cycle and epidermal homeostasis. Hair follicle morphogenesis initiates during embryogenesis and ceases approximately 2–3 weeks postnatally, whereby the ensuing post-morphogenic hair cycle marks a new anagen stage (Figure 4A). Using immunostaining, we monitored Ddit4 protein expression during transitioning hair follicular and epidermal keratinocytes through morphogenic and post-morphogenic stages within WT and VDR^−/−^ mice maintained on a rescue diet. Catagen is a transitional apoptosis-mediated involution stage that signals the end of the active growth of hair. Importantly, the VDR is selectively expressed in hair follicle keratinocytes during late anagen and catagen stages, which reflects the reduced period of proliferation and elevated differentiation of follicular cells [39]. At day 14 during the first postnatal early catagen phase, we observed distinct Ddit4 staining patterns between WT and VDR^−/−^ hair follicles and epidermis derived from littermates (Figure 4B). Both types of mice showed Ddit4 expression in the inner root sheath (IRS) of the hair follicle and moderate levels within the interfollicular epidermal regions (Figure 4B). However, there was slightly higher, atypical Ddit4 expression in VDR^−/−^ follicles. These punctate regions of increased Ddit4 expression within VDR^−/−^ follicles were observed in nearly every follicle within superbasal sections at day 14 (Figure 4(BIII–VI)). Given the stress inducible role of Ddit4, we classified these areas as stress compartments (SC) within VDR-deficient follicles. These results suggest that VDR-deficient hair follicles that are transitioning between catagen-telogen exhibit increased cellular stress.

By day 29, initiation of post-morphogenic anagen in the hair follicle is characterized by increased cell proliferation in the follicular epithelium below the activated bulge. During anagen in WT skin, Ddit4 expression was substantially reduced within the hair follicle and interfollicular regions compared to day 14 (Figure 4C), suggesting a reduction of its growth-inhibitory functions. Despite this reduction, there was more residual Ddit4 within the interfollicular epidermis compared to the follicle. At this stage, VDR-null hair follicles are degenerated and exhibit an abnormal morphology depicted by follicular dystrophy, IRS hyperplasia and massive dilation of the junctional canal consistent with previous work [17]. In contrast to normal hair follicles, VDR-null follicles exhibit increased and variegated expression of Ddit4 throughout the dystrophic follicular epithelium and interfollicular regions. There was a statistically significant increase in normalized average fluorescent intensity (VDR^−/−^: 538 ± 31 a.u.; WT: 74 ± 11 a.u., *n* = 8 follicles, *p* < 0.01) within the bulb region of VDR^−/−^ hair follicles when compared to WT samples at this stage. We also found that the utricules (u), i.e., the epidermal portion of abnormal hair follicles, also expressed Ddit4 in the lining epithelium. By day 48, the hair follicles of normal animals remain in the resting telogen phase and are kept dormant for another five weeks. The epidermis at this stage also expresses major factors of differentiation [4]. In WT animals, the epidermis and follicular epithelium uniformly expressed increased levels of Ddit4 (Figure 4D), presumably to help maintain a growth-inhibiting, dormant state. Furthermore, dermal fibroblasts in WT skin also expressed Ddit4. On the other hand, VDR-null mice at day 48 exhibit epidermal hyperplasia and increased epidermal corneocytes (c), suggesting abnormal shedding of cornified material. Remarkably, Ddit4 expression was absent within VDR-null skin at telogen. Ddit4 expression was suppressed throughout the epidermis, remaining non-exogenic follicular epithelium and within the dermis as well. Furthermore, there was increased dermal cellularity in VDR^−/−^ skin, inversely correlated with the level of Ddit4 (Figure 4D).

### 2.5. The VDR Does Not Regulate Ddit4 within Bulge Keratinocyte Stem Cells

Besides epidermal and follicular keratinocytes, the cutaneous niche consists of many different cell types, including bulge keratinocyte stem cells (KSCs). It was recently shown that hair follicle growth and stem cell exhaustion in mice are linked to mTOR dysregulation as a means to maintain genetic integrity of the stem cell population [40,41]. We therefore hypothesized that the unliganded VDR maintains proper *Ddit4* levels, and hence mTOR function, within KSCs. We compared *Ddit4* transcript levels under unliganded conditions within 29-day-old KSCs, a period in which KSCs are activated (Figure 4C). We isolated living bulge KSC populations from anagen hair follicles of WT and VDR^−/−^ littermates using FACs purification according to the established markers α6^hi^ and CD34^+^ [42]. Based on these preparations, we observed no difference in *Ddit4* message levels between WT and VDR^−/−^ KSCs. These results suggest no relationship between the VDR and mTOR signaling towards possible stem cell exhaustion and no effect on the hair loss phenotype, raising the question of primary defects in progenitor cells instead.

### 2.6. Impaired Ddit4/*Ddit4* and Krt10/*Krt10* Expression in the Neo-Epidermis of Wounds from VDR^−/−^ Mice

**Figure 5:**
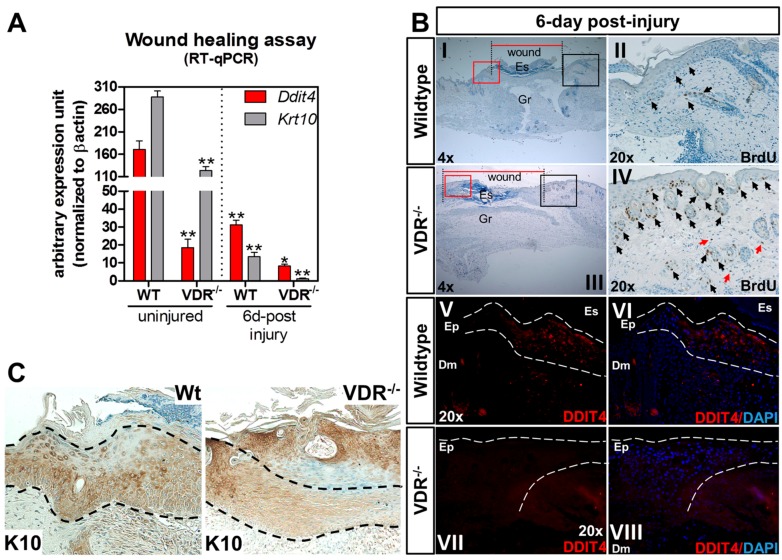
Reduction of Ddit4 in the re-epithelized wounds of VDR^−/−^ animals (column 1.5). (**A**) RT-qPCR analysis of the wound repair process from excised wounds. For uninjured samples, comparisons were made between genotypes. Six days following injury, sample comparisons were made between the respective uninjured genotypes. One-way ANOVA at an α = 0.05 (95% confidence interval) and Tukey’s multiple comparison post-tests were utilized. Significance is denoted with asterisks: * *p* < 0.05, ** *p* < 0.01 (*n* = 4 samples per genotype and time point); (**B**) Representative slides showing 5-bromo-2′-deoxyuridine (BrdU) immunohistochemical (**I**–**IV**) and Ddit4 immunofluorescence (**V**–**VIII**) staining of WT and VDR^−/−^ skins six days following injury. Wound closure is depicted by the horizontal red bars. Black boxed areas in the 4× slides represent the BrdU-labeled magnified regions (**II** and **IV**). Red-boxed areas represent the Ddit4-labeled magnified regions (**V**–**VII**). In the BrdU slides, black arrows depict proliferating epidermal and follicular keratinocytes. Red arrows depict proliferating dermal fibroblasts. In the Ddit4 panels, the dotted white line marks the re-epithelialized area. Nuclei are marked in **blue** with 4′,6-diamidino-2-phenylindole (DAPI), while Ddit4 is labeled in **red**. ES: eschar; GR: granulation tissue; EP: epidermis; DM dermis; (**C**) Krt10 immunostaining within the neo-epidermis (demarcated by dashed lines) six days following injury.

The cause for delayed onset of cutaneous wound closure in global VDR-null mice is unclear. Based on this, we studied the association between Ddit4 and VDR during the cutaneous wound repair process. Mice aged 47 days mice were subjected to 3.5 mm trunk punch biopsies and tissues were harvested and processed for RT-qPCR, BrdU (5-bromo-2′-deoxyuridine) labeling, and immunocytochemistry six days after injury. First, we monitored and compared *Ddit4* and *Krt10* (keratin 10) message levels between WT and VDR^−/−^ animals. Krt10 is an early marker of differentiating daughter cells in the stratum basale which faces the epidermal surface. We confirmed the lower *Ddit4* message levels within uninjured VDR^−/−^ epidermis, which was accompanied by a significant reduction in *Krt10* mRNA compared to uninjured WT tissue (Figure 5A). Relative to uninjured skin, there was a significant decrease in both *Ddit4* and *Krt10* message levels in WT wounds six days post-injury, representing the early-intermediate phase of the re-epithelialization process. Compared to WT wounds six days after injury, *Ddit4* and *Krt10* mRNA levels were further attenuated in VDR^−/−^ wounds. Histological analysis of wound closure showed larger wound openings (Figure 5B, red lines) in VDR^−/−^ mice after six days. Interestingly, within VDR^−/−^ injured skin, in vivo BrdU labeling revealed an increased number of proliferating epidermal and follicular keratinocytes compared to WT samples at the wound edges, marking a potential reserve of cells impaired in the re-epithelialization process (Figure 5(BII,IV); representative black box). In both genotypes, BrdU also labeled damaged hair follicles. Particular to VDR^−/−^ tissue were BrdU-positive proliferating dermal fibroblasts (red arrows). Next we monitored Ddit4 and Krt10 expression with immunohistochemistry in the newly restored epidermis six days after injury. We observed moderate expression of Ddit4 within the newly re-epithelized wound edge in WT animals (Figure 5(BV,VI); representative red box). In contrast, there was a comparable decrease in Ddit4-positive cells present within the neo-epidermis in VDR^−/−^ animals (Figure 5(BVII,VIII); representative red box). In WT neo-epidermis, there were diffuse cells with high Krt10 immuno-reactivity in contrast to VDR^−/−^ wounds (Figure 5C). Overall, these findings suggest that the loss of Ddit4/Krt10-positive cells in wounded VDR^−/−^ animals contributes to the impaired re-epithelialization process.

### 2.7. Ddit4-Deficient Mouse Embryonic Fibroblasts Are Resistant to the Pro-Differentiation Actions of Vitamin D

**Figure 6:**
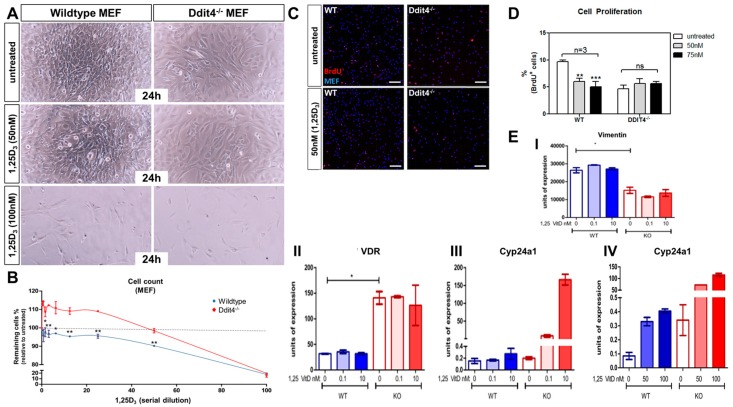
Ddit4^−/−^ mouse embryonic fibroblasts are resistant to the pro-differentiation actions of vitamin D (1 column). (**A**) Representative images of WT and Ddit4^−/−^ mouse embryonic fibroblasts (MEFs). MEFs were untreated or treated with a serial dilution of 1,25D_3_ (0.39–100 nM) for 24 h; (**B**) Cell counts performed after 1,25D_3_ treatment to calculate the proliferation % relative to the untreated sample. One-way ANOVA at an α = 0.05 (95% confidence interval) and Tukey’s multiple comparison post-tests were utilized. Significance is denoted with asterisks: * *p* < 0.05, ** *p* < 0.01 (*n* = 3 experiments per conditions and genotype); (**C**) Representative BrdU-labeled MEFs shown after 1,25D_3_ treatment for 12 h. DAPI-positive cells were marked using the Imaris (Bitplane) software to perform quantitative analysis. Bar = 50 µm; (**D**) Quantification of BrdU-labeled MEFs after 1,25D_3_ treatment. Significance is denoted with asterisks: ** *p* < 0.01, *** *p* < 0.001 (*n* = 3 experiments per conditions and genotype), ns (not significant); (**E**) Quantitative PCR analysis of *Vimentin* in Wt and KO MEFs after 6 h with or without vitamin D, * *p* < 0.05 (**I**). Quantitative PCR analysis of MEFs treated or untreated with low or high concentrations of 1,25D_3_ for 6 h (**II**–**IV**). Increased expression of *Vdr* (**II**) and induction of *Cyp24a1* (**III**,**IV**) within Ddit4^−/−^ MEFs. (*n* = 3 preparations of each cell line).

Although the initial data suggests a functional association between VDR and Ddit4, the impact of Ddit4 on vitamin D function is unclear. As Ddit4 is expressed in dermal fibroblasts and dysregulated in VDR^−/−^ skin (Figure 4D), mouse embryonic fibroblasts (MEFs) derived from Ddit4^−/−^ and WT animals (kindly provided by Leif W. Ellisen) were tested for any differences in classic vitamin D anti-proliferative responses. We observed more spindle-shaped cells, characteristic of mitotic cells, in Ddit4-deficient MEFs even following 50 nM 1,25D_3_ treatment (Figure 6A, right panels). Measurement of cell proliferation in WT MEFs showed that even at the lowest concentration range of 1,25D_3_, cell proliferation was inhibited compared to the baseline (dotted line) (Figure 6B). At 50 nM 1,25D_3_, there was an approximate 10% decrease in cell proliferation in WT samples, but no effect in Ddit4^−/−^ MEFs. It was only at the highest level (100 nM) of 1,25D_3_ that Ddit4*^−/−^* MEFs exhibited comparable effects on cell number to WT cells (Figure 6A,B), possibly succumbing to apoptosis [43]. Next, we performed BrdU-incorporation studies using MEFs treated with 50–75 nM 1,25D_3_ (Figure 6C,D), and observed statistically significant decreases in BrdU incorporation within WT, but not Ddit4^−/−^ MEFs. The fibroblast maturation marker vimentin was also decreased in Ddit4^−/−^ MEFs when compared to WT samples (Figure 6(EI)). Lastly, to gain insight into signaling events, we performed quantitative real-time PCR analysis to monitor *Vdr* expression and induction of Cyp24a1 (Figure 6(EII–IV)). Interestingly, Ddit4^−/−^ MEFs expressed more *Vdr* compared to normal MEFs (Figure 6(EII)). As a result, there was a concomitant increase in induction of *Cyp24a1* in Ddit4^−/−^ MEFs (Figure 6(EIII,IV)). These results suggest that despite the increase in Vdr and its signaling capacity in MEFs lacking Ddit4, vitamin D is unable to differentiate these cells due to specific defects in the DDIT4 signaling cascade. In conclusion, Ddit4-deficient MEFs were resistant to low-to-moderate vitamin D treatments, and Ddit4 is a downstream effector of liganded VDR actions to promote cellular differentiation within fibroblasts.

## 3. Discussion

### 3.1. Insights into Ddit4-VDR

Perturbation of VDR expression in humans and mice is associated with alopecia. The results reported here therefore provide novel insights into biological and clinical applications of vitamin D and skin function. Our data show that the mTOR inhibitor, *Ddit4*, is a direct transcriptional target of VDR in epidermal keratinocytes. Regulation of mTOR activity is a major component of “checks and balances” of energy homeostasis within a cell. Ddit4, a mitochondria-resident protein, represses mTOR signaling by activating TSC2 (tuberin), a guanosine triphosphate (GTP) hydrolyzing enzyme (GTPase) activating protein (GAP), which then stimulates the small GTPase Ras homolog enriched in brain (Rheb) in its GDP-bound form to inactivate mTOR [25]. Dysregulation of mTOR signaling can lead to a plethora of diseases including cancer and metabolic disorders. Studies have shown that 1,25D_3_ or its analogues can suppress tumor cell growth by upregulation of Ddit4 in various cancer cell model systems [44]. Suppression of mTOR via Ddit4 activation results in attenuation in both cell size and growth rate, comparable to that observed after nutrient and growth factor deprivation [45]. Conversely, Ddit4 levels are mitigated under growth-like conditions [27]. The importance of Ddit4 in regulating cytoprotection and survival is highlighted in the Ddit4^−/−^ mouse line that is associated with enhanced mTOR activity [25]. DDIT4^−/−^ mice are known to be resistant to a diverse set of stress conditions such as those caused by oxidative stress in the retina, tobacco smoke-induced emphysema, steroid-induced atrophy in the skin and apoptosis of lung epithelial cells caused by ceramide [46,47,48]. Mouse *Ddit4* resides in a genomic region on chromosome 10 (10: 59316668-74913026), which forms the only syntenic cluster on human chromosome 10 (10: 53435340-73103214), emphasizing its conservation, genomic and functional importance. In addition, previous studies found that simultaneous loss of Drosophila *scylla* and *charybdis*, which are homologs of the human *DDIT4* and *DDIT4-like* genes, generated flies that showed mild overgrowth [49]. In contrast, enhanced expression of Ddit4 can promote apoptotic cell death or terminal differentiation in certain cell types [50], including VDR-deficient follicular epithelial cells observed in our studies.

### 3.2. Hair Follicle Defects in VDR-Deficient Animals

Our data support the notion that abnormalities in Ddit4 signaling disrupt follicular energy homeostasis to affect follicular integrity in VDR^−/−^ animals. It was previously reported that the inability of VDR-deficient animals to initiate a new post-morphogenic hair cycle was due to primary defects within KSCs [13]. Additional findings observed no change in differentiation markers of VDR-deficient neonatal keratinocytes in culture, concluding that the mutant follicular keratinocytes during the morphogenic period are normal [5]. In contrast, the findings of this study and others have shown that there exist defects within the follicular epithelium of VDR^−/−^ mice during the catagen-to-telogen morphogenic period [17]. In this regard, it has been shown that the failure of the follicular epidermis to maintain the hair follicle in VDR-deficient animals likely represents the compromised adhesion and motility capacity of surface cells along the follicle at the onset of anagen, and not due to functional defects within label-retaining KSCs [14,19]. These defects can further compromise lineage progression and differentiation status of follicular keratinocytes lacking a functional VDR. Furthermore, we observed no differences in *Ddit4* expression within VDR^−/−^ KSCs, suggesting that Ddit4-related defects reside either in the hair germ or more in differentiated progenitor cells. It is well known that mTOR activation propagates metastasis and matrix-stimulated cell migration, and treatment with mTOR inhibitors such as rapamycin can block cell motility under numerous experimental conditions (reviewed in [51]). There are also several examples how primary defects of the strictly regulated morphogenic catagen-telogen transition can lead to failed initiation of the ensuing anagen hair cycle, much like that observed in VDR^−/−^ animals [52,53]. Overall, our findings may have uncovered a critical cog—mTOR signaling—in the full understanding of how hair follicles become impaired and degenerate in VDR^−/−^ animals.

**Figure 7:**
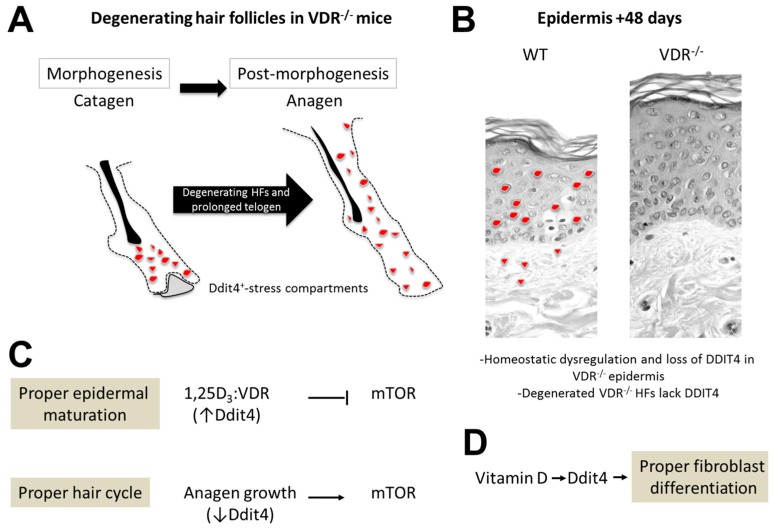
Early stress in VDR^−/−^ hair follicles and epidermal regulation of Ddit4 by the VDR (column 1.5). Schema depicting the major findings. (**A**) VDR^−/−^ hair follicles (HFs) exhibit DDIT4^+^ stress compartments, marked in red, during morphogenesis which prolongs the telogen phase; (**B**) The VDR regulates Ddit4 expression during epidermal homeostasis and wound healing responses. By day 48, VDR^−/−^ hair follicles have degenerated resulting in loss of Ddit4expression; (**C**) Proposed functional role of Ddit4 and mTOR during epidermal maturation and hair growth. During epidermal maturation, VDR signaling induces *Ddit4* expression to block mTOR cascades. During the initiation of anagen, Ddit4 levels are suppressed in order to stimulate the mTOR growth-promoting cascade; (**D**) Proposed involvement of DDIT4 in regulating the pro-differentiation effects of vitamin D in embryonic fibroblasts.

To date, it is unclear if the stress compartments identified in VDR^−/−^ morphogenic follicles are formed due to systemic and/or local stress stimuli. As a number of sensing cues impinge on the Ddit4-mTOR signaling pathway, it will require additional efforts to identify the specific cues within the VDR^−/−^ follicles. It is commonly known that damaged hair follicles endure premature catagen initiation to inhibit proper hair follicle growth [54], further supporting our findings and suggesting precocious and prolonged catagen-to-telogen transition in VDR-deficient follicles. This speculation is supported by a recent study which performed RT-qPCR analysis on plucked VDR^−/−^ hair follicles during morphogenetic days 13 and 15 [17]. In the study, at day 13 there was a significant increase in pro-apoptotic *CASP3* (caspase 3) transcripts in VDR^−/−^ follicles compared to controls, and at day 15 there was a reported decrease in *Shh* (sonic hedgehog) message levels. Epithelial Shh is a major driver of hair follicle morphogenesis, and its decrease in VDR^−/−^ follicles hints at perturbations in the process. Furthermore, it is unclear if the elevated Ddit4 level in VDR^−/−^ follicles is a harbinger of apoptosis activation [17], as there is a clear link between persistent Ddit4 activation with programmed cell death [55]. Lastly, we are unclear whether the Ddit4-positive stress compartments are associated with clearance of dysfunctional cellular debris, as DDIT4 upregulation and mTOR suppression are positively correlated with autophagocytosis [56]. Overall, we speculate that follicular degeneration in VDR-null animals does not reflect defects in the VDR-Ddit4 axis within KSCs, rather in the cells that make up the cycling portion of the hair follicle (Figure 7).

### 3.3. Epidermal Wounding Defects

The defect in VDR^−/−^ hair follicles highlights the dichotomy which exists in the epidermis (Figure 7). Our data suggests that within uninjured epidermal keratinocytes, both in vitro and in vivo, the VDR regulates *Ddit4* message levels in a concentration and age-dependent manner, respectively. By day 48 the epidermis normally expresses high levels of differentiation markers, yet animals void of VDR associate with reduced epidermal differentiation [17,57]. This is consistent with reduced levels of Ddit4 within skin of VDR^−/−^ mice. This finding is also consistent with increased Shh in the epidermis of VDR^−/−^ mice [57], which can induce a basal-like phenotype as well as basal cell carcinomas in the skin. Thus, we speculate that the VDR maintains epidermal homeostasis via direct transcriptional control of *Ddit4* over time.

Skin wounding triggers an acute inflammatory response with the innate immune system contributing both to protection against invasive organisms and invasion of inflammatory cells into the wounded area. These cells release a variety of cytokines and growth factors that stimulate the proliferation and migration of dermal and epidermal cells to close the wound. Mice globally lacking the VDR or the enzyme CYP27B1 exhibit decreased lipid content of the lamellar bodies leading to a defective permeability barrier [58], and a defective response of the innate immune system to invading infections acting through dermal TGF-β signaling [2]. We observed delayed wound closure in the VDR^−/−^ mice, which is consistent with most reports [3,14,59]. This is in contrast to one study that showed no difference in wound closure of VDR^−/−^ animals [2], which may reflect differences in the age of the animals and/or severity and conditions of the wounds. Regardless, studies investigating “epithelial-specific” ablation of the VDR resulted in delayed wound closure attributed to impaired β-catenin signaling within epidermal stem cells [3]. Recently, epithelial-specific ablation of phosphatase and tensin homolog *Pten* and tuberous sclerosis 1 *Tsc1* (both inhibitors of mTOR) has shown that mTOR activation can dramatically increase epithelial cell migration and cutaneous wound closure [40]. Although we observed the opposite phenomena, one could argue for global VDR ablation having systemic effects on the immune system that impedes the subsequent steps of the healing process, such as re-epithelialization.

Fibroblasts also migrate into the wounded area and proliferate to deposit a provisional extracellular matrix consisting of reforming granulation tissue. Keratinocytes migrate across the injured dermis above the provisional matrix and begin to proliferate. By 3–10 days after injury, the wound is filled with granulation tissue, and fibroblasts are recruited to the wound by growth factors from macrophages. Fibroblasts then *trans*-differentiate into myofibroblasts, leading to wound contraction and immature collagen deposition assisting in wound closure. At this stage, the apical wound portion is overlaid with a neo-epidermis associated with fibroblasts. At six days after wounding we observed sporadic Ddit4-postive cells within the re-epithelized wound within the neo-epidermis in WT animals. The identity of these cells is unclear, and may signify the transitioning (myo)fibroblasts during the healing process or keratinocytes. At this stage the levels of both *Ddit4*/Ddit4 and *Krt10*/Krt10 decreased relative to uninjured tissue representing the development-like regenerative steps of healing. In contrast, VDR^−/−^ neo-epidermis did not harbor any Ddit4-positive cells, suggesting loss of or delayed formation and recruitment of this specialized subset of regenerative cells during the healing process. Importantly, we observed primary ligand resistance to VDR signaling within Ddit4^−/−^ MEFs, suggesting potential dermal fibroblastic regulation and defects in VDR^−/−^ animals as well.

## 4. Materials and Methods

### 4.1. Animal Maintenance

VDR^−/−^ animals (B6.129S4-Vdr^tm1Mbd/J^) with targeted ablation of the second zinc finger were purchased from the Jackson Laboratory (JAX: 006133, Bar Harbor, ME, USA). Animal studies were approved by the institutional animal care and use committee (First Affiliated Hospital of Chongqing Medical University; SYXK2012-0001, January 2015). Animals were kept in a clean (virus- and parasite-free) facility under a 12-h light, 12-h dark cycle on a diet enriched with calcium (2%), phosphorus (1.25%) and lactose (20%) to prevent hyperparathyroidism, rickets and osteomalacia, but not alopecia [16].

### 4.2. Mouse Puncture Assay

Male 48-day-old littermate mice (*n* = 4 mice per time point) were anesthetized and received a 3.5 mm biopsy skin punch on each side of the trunk. Six days after puncture, re-epithelialization was monitored by harvesting tissue in 10% buffered formalin. Paraffin embedded wounds were processed for immunohistochemistry. For reverse transcription quantitative PCR (RT-qPCR) analysis, wounds (*n* = 4 per genotype and time point) were excised, trimmed and then processed using the RNeasy Plus Universal Mini Kit (Qiagen, Gaithersburg, MD, USA).

### 4.3. Chromatin Immunoprecipitation (ChIP)

We performed the ChIP assays using ChIP-IT^®^ Express Chromatin Immunoprecipitation Kits from Active Motif. Primary keratinocytes were treated for 15 min with 10 nM 1,25D_3_ (Biomol, Plymouth Meeting, PA, USA) reconstituted in absolute ethanol. Chromatin was cross-linked with 1% formaldehyde, quenched with glycine and processed in 1% sodium dodecyl sulfate (SDS) cell lysis buffer. Samples were enzyme-treated to yield fragmented chromatin. Chromatin samples were incubated with 10 µg ChIP-grade anti-RNA polymerase II (ab26721; Abcam, Boston, MA, USA) and anti-VDR (sc-1008x; Santa Cruz Biotechnology, Santa Cruz, CA, USA) antibodies. Quantitative PCR (qPCR) was performed with primers flanking putative vitamin D response element (VDRE) consensus sequences in the regulatory region of mouse Ddit4 (based on the GRCm38 mouse assembly; available on: https://www.ncbi.nlm.nih.gov/genome/52). Primers were designed using Primer3 (bioinfo.ut.ee/primer3-0.4.0/primer3). Ddit4 target: Ddit4-1: (forward) 5-ttcccatccttttgcagttc-3, (reverse) 5-ccactgcccaatttcatctt-3; Ddit4-2: (forward) 5-tcagggtcccagtgtcctac-3, (reverse) 5-caattcaatggaacccagga-3; and Ddit4-3: (forward) 5-ggtacctttctcccctgctc-3, (reverse) 5-ctctcccctcgccttagc-3. Control primers: OC (osteocalcin): (forward) 5-caggggcagacactgaaaa-3, (reverse) 5-aggagactgccaggttctga-3; VDR-S1 (VDRE site): (forward) 5-gtagccatccatgtggcttt-3, (reverse) 5-ccagacggaagcctagagaa-3; and non-specific control (calponin) based on [60]. The non-specific control was used to normalize for DNA content and to calculate the relative enrichment of the regulatory regions according to the formula of Livak and Schmittgen [61] and presented as fold enrichment. Samples were run on a 1.5% tris-acetic acid- ethylenediaminetetraacetic acid (TAE) agarose gel for visualization.

### 4.4. In Vivo/In Vitro 5-Bromo-2′-deoxyuridine (BrdU) Labeling and Immunocytochemistry

For cell proliferation studies, wounded animals were injected intraperitoneally with 5-bromo-2′-deoxyuridine (BrdU) at 0.25 mg/gram (Roche) three hours prior to the time that the animals were sacrificed. Wounds were excised, fixed, and processed for immunocytochemical analysis. Proliferating cells were detected using a BrdU labeling and detection kit (Roche, Indianapolis, IN, USA) according to the manufacturer′s instructions. For in vitro studies, sterile filtered 10 µM BrdU was prepared in media. After 12 h of vitamin D treatment, cells were washed with phosphate buffered saline (PBS), and replaced with the BrdU solution for a 2 h labeling period. Cells were fixed and then detected as above. Cell counting analysis was performed using the “spot” function on the Imaris (version 8.4, Bitplane, Concord, MA, USA) software.

### 4.5. Fluorescence-Activated Cell Sorting of Hair Follicle Stem Cells

Skin from 29-day-old wildtype (WT) and VDR^−/−^ littermates was used to harvest hair follicle stem cells. The purification of bulge stem cells from mice was performed using established expression markers α6^hi^/Cluster of differentiation 34 (CD34^+^) [12]. The antibodies used were: Anti-Integrin α6 phycoerythrin (PE) conjugated, ab95703 (Abcam); anti-CD34, ab8158 (Abcam); and Alexa Fluor^®^ 488 (Thermofisher, Waltham, MA, USA) secondary antibody at ~1 µg antibody per 10^6^ cells. The double-labeled cell suspensions were placed in FACS tubes on ice for sorting on a BD FACSCalibur™ (BD Biosciences, San Jose, CA, USA). BD Falcon tubes containing 50% chelex-treated FBS in PBS were used to collect double-labeled KSCs. Using four sets (*n* = 4 experiments per genotype) of four 29-day-old postnatal mice, we enriched KSCs for RT-qPCR analysis. KSCs were further collected in the appropriate RNA stabilization reagent supplied in the RNeasy mini kit (Qiagen).

### 4.6. Immunofluorescent and Immunohistochemical Labeling of Skin

Skin tissues were obtained from the lower dorsal region. Wounds were excised and fixed in 10% formalin/PBS, processed and embedded vertically in paraffin. Tissues sections were mounted on microscope slides and processed for immunofluorescence staining with detergent (0.1% triton X). DDIT4 labeling was detected using an DDIT4 antibody (Abcam, ab106356) at 1:200 dilution followed by secondary staining with Alexa Fluor^®^ 594 (Thermofisher). Slides were mounted in Vectashield^®^ mounting medium with DAPI (Vector Laboratory). Immunohistochemistry was performed using anti-K10 (Abcam, ab9026) at 1:100 dilution with HRP-conjugated secondary antibody and DAB. Slides were imaged using a FV1000 (Olympus, Center Valley, PA, USA) confocal microscope. Control slides were included by negating primary antibodies or substituting with pre-immune sera (data not shown). For confocal imaging, a series of three-dimensional “*z*-axis” image projections of follicular axial depths were obtained in XYZ scan mode set to 1 µm/slice and a sample speed of 12.5 µs/pixel. All other parameters (e.g., pinhole diameter, gain, laser intensities) were kept constant during imaging. The fluorescence intensity was never saturated (max. 4096 intensity level) during imaging. The series of projected “*z*-axis” images were used to calculate average fluorescent intensity profiles per 594 nm channel using the freehand analysis tool in the Fluoview software v. 4.1 (Olympus). The proximal bulb region of Ddit4-stained hair follicles were averaged per image, as well as follicles of negative controls averaged per image to generate average background fluorescence intensities. Ddit4 levels in the bulb were relatively compared and background corrected between for eight individual follicles per genotype.

### 4.7. Ddit4^−/−^ Mouse Embryonic Fibroblasts (MEFs) and Cell Count Measurement

Ddit4^−/−^ and wild type MEFs were generously provided by Leif W. Ellisen as previously described [25], and maintained in Dulbecco’s Modified Eagle’s medium (DMEM)/10% fetal bovine serum, Pen/Strep. Cells were maintained at 37 °C in a 95% air/5% CO_2_ atmosphere. Cells were plated in 24-well tissue culture plates at 2 × 10^4^ cells per cm^2^ and then replaced with fresh media and 1,25D_3_. Cells were trypsinized and counted 24 h later using an automated cell counter (Countess II FL, Thermofisher). Three sample preparations were made per condition in order to calculate the % of cells remaining (*n* = 3).

### 4.8. mRNA Reverse Transcription Quantitative PCR (RT-qPCR) Analysis

Total RNA was purified using the RNeasy mini kit (Qiagen). cDNA was synthesized from 300 ng total RNA by SuperScript Reverse Transcriptase III (Invitrogen, Carlsbad, CA, USA) using random hexamers. RT-qPCR analysis was performed with a Stratagene MX-3005P instrument utilizing TaqMan system reagents from ABI, and target genes were normalized to ATCB (β-actin) expression. TaqMan^®^ assays used: (1) Control ATCB, β-actin (VIC^®^-labeled), assay ID 4352341E; (2) 1,25-dihydroxyvitamin D_3_ 24-hydroxylase (Cyp24a1, FAM™-labeled), assay ID 4331182; (3) Ddit4 (FAM™-labeled), assay ID 4331182; and (4) Krt10 (FAM™-labeled), assay ID 4331182. All reactions were performed in triplicate experimental conditions. Data are presented as comparable arbitrary expression units.

### 4.9. Primary Neonatal Keratinocytes and mVDR Transient Transfection

Primary keratinocytes were harvested from neonatal pups aged 2–3 days using a trypsin floating method. Briefly, the pups were skinned and then floated on 0.05% trypsin (Sigma, St. Louis, MO, USA) at low temperature for 12 h. The epidermal sheets were harvested, minced, and then agitated with a stir bar in a keratinocyte growth medium on ice for 1 h. The medium consisted of: calcium and magnesium-free Eagle’s Minimal Essential Medium (Gibco, Carlsbad, CA, USA), FBS with reduced calcium, 2 ng/mL human recombinant epidermal growth factor (EGF) (Novoprotein, Summit, NJ, USA) supplemented with 1× penicillin–streptomycin (Gibco). Calcium in the serum was removed by treating FBS with a chelating resin, Chelex^®^100 (Bio-Rad Laboratories, Hercules, CA, USA). Calcium concentration was adjusted to 0.05 mM by adding calcium chloride solution. The cell suspension was then filtered using a 40 micron mesh and were seeded (4 × 10^4^ cells/cm^2^) in tissue culture plates pre-coated with type I collagen (Gibco, R-011-K). Cultures were incubated in 8% CO_2_ and 92% humidified atmosphere at 34 °C, and medium was changed every 2–3 days. The untagged mouse vitamin D receptor expression vector (pCMV6) was purchased from Origene (BC006716). Transfection of primary keratinocytes was conducted using the BioT reagent (Biolands) and protocol.

## 5. Conclusions

In conclusion, we show that Ddit4 marks early stress compartments within VDR^−/−^ hair follicles which initiate during the morphogenic period. Importantly, we implicate Ddit4 as a functional component of growing anagen follicles during the hair cycle. Ddit4 is also a direct transcriptional target of the VDR within epidermal keratinocytes, highlighting its ligand-dependent genomic role during vitamin D signaling. VDR regulates Ddit4 during epidermal homeostasis and the wound repair process, namely the proper differentiation and stratification of the neo-epidermis post injury.

## Abbreviations:

VDR	Vitamin D receptor
Ddit4	DNA damage-inducible transcript 4
VDRE	Vitamin D response element
KSC	Keratinocyte stem cell
mTOR	Mechanistic/mammalian target of rapamycin
